# Compositional Associations of 24‐h Movement Behaviors With Depressive and Anxiety Symptoms in Middle‐Aged Adults

**DOI:** 10.1155/da/6881070

**Published:** 2026-04-28

**Authors:** Clarence Tan, Maisa Niemelä, Marjo Seppänen, Anna-Maiju Leinonen, Vahid Farrahi

**Affiliations:** ^1^ Research Unit of Health Sciences and Technology, University of Oulu, Oulu, Finland, oulu.fi; ^2^ Centre for Wireless Communications, University of Oulu, Oulu, Finland, oulu.fi; ^3^ Research Unit of Population Health, University of Oulu, Oulu, Finland, oulu.fi; ^4^ Research Unit of Geography, University of Oulu, Oulu, Finland, oulu.fi; ^5^ Department of Sports and Exercise Medicine, Oulu Deaconess Institute Foundation sr, Oulu, Finland; ^6^ Institute for Sports and Sport Science, TU Dortmund University, Dortmund, Germany, tu-dortmund.de

**Keywords:** accelerometer, cohort study, mental health, physical activity, sedentary behavior

## Abstract

**Background:**

Physical activity could reduce the risk of depression and anxiety. A 24‐h day includes time spent sleeping, in sedentary behavior (SB), or engaging in physical activities. However, the joint and combined associations of these 24‐h movement behaviors with depressive and anxiety symptoms remain unclear.

**Aim:**

To investigate the compositional associations of 24‐h movement behaviors with depressive and anxiety symptoms in a population‐based sample of middle‐aged adults.

**Methods:**

The study population (*N* = 4490) comprised of participants from the Northern Finland Birth Cohort 1966 (NFBC1966). Over 14 consecutive days, movement behaviors—SB, light physical activity (LPA), and moderate‐to‐vigorous physical activity (MVPA)—were recorded using a hip‐worn accelerometer and were combined with self‐reported sleep duration to obtain the 24‐h time‐use composition. Three different screening tools—Beck Depression Inventory‐II (BDI‐II), Generalized Anxiety Disorder‐7 (GAD‐7), and Hopkins Symptom Checklist‐25 (HSCL‐25)—were utilized to assess the severity of depressive and anxiety symptoms. Compositional linear regression and time reallocation analysis were performed to examine the associations of 24‐h movement behaviors with depressive and anxiety symptoms.

**Results:**

Compositional 24‐h movement behaviors were significantly associated with depressive and anxiety symptoms. Higher MVPA relative to other components of 24‐h movement behaviors was associated with significantly lower depressive and anxiety symptoms. A 30‐min reallocation of time from SB to MVPA was associated with 9.0% (95% confidence interval [CI]: 6.3%, 11.6%) lower depressive symptoms and 4.5% (95% CI: 2.1%, 6.9%) lower anxiety symptoms assessed with BDI‐II and GAD‐7 tools, respectively. Similar patterns of associations were observed in the time reallocation analysis when depressive and anxiety symptoms were assessed with the HSCL‐25 screening tool.

**Conclusion:**

Each component of 24‐h movement behaviors was associated with depressive and anxiety symptoms. Among the component parts of 24‐h movement behaviors, MVPA was most strongly associated with lower depressive and anxiety symptoms, whereas LPA only showed marginal favorable benefits.

## 1. Introduction

Depression and anxiety are common mental disorders that are highly ranked as the leading cause of disability worldwide, affecting approximately 4%–5% of adults globally [1–3]. People with depression tend to have other mental health conditions, given that depression and anxiety disorders are highly comorbid with each other [4]. Numerous studies have highlighted the importance of physical activity for depression and anxiety [5–8]. Higher levels of physical activity are associated with lower depressive and anxiety symptoms [5, 6] and are inversely related to incident depression and anxiety [9]. Physical activity is also effective for reducing depressive and anxiety symptoms [10], especially among adults who meet the World Health Organization’s current guidelines of at least 150 min of moderate‐intensity physical activity per week [11].

Sedentary behavior (SB), on the other hand, has been shown to associate with higher risk of depressive and anxiety symptoms [12, 13], and reducing it can improve mental health [14]. Sleep is also essential for better overall mental health [15], and sleep duration is associated with depressive and anxiety symptoms in adults [16, 17]. Although the relationship between long sleep duration and mental health is inconclusive, short sleep duration (<6 h) has been shown to negatively associate with mental health, such as increased risk of depression and anxiety [15].

A complete 24‐h day consists of four main components, including sleep, SB, light physical activity (LPA), and moderate‐to‐vigorous physical activity (MVPA) [18, 19]. Evidence indicates that each of these movement behaviors is associated with mental health outcomes [7, 8, 12–14], suggesting that these behaviors may collectively influence these outcomes [18, 19]. However, previous studies on the health associations of movement behaviors typically examined the associations of physical activities, SB, and sleep in isolation, with no or partial consideration for the other movement behaviors within the 24‐h daily cycle, often using traditional regression methods [18–20]. Recent research has recognized that improper or partial accounting for any component of 24‐h movement behavior could be problematic and lead to biased associations with health outcomes [18, 19, 21]. For example, several studies did not consider sleep when investigating the associations between physical activity, SB, and mental health outcomes [22–24]. This impartial accounting implicitly assumes that changes in waking behaviors occur independently of sleep, despite evidence of potentially complex interactions among physical activity, SB, and sleep in their associations with health outcomes [18–21].

Over the past few decades, observational and cohort studies have increasingly relied on wearable accelerometers, enabling continuous moment‐by‐moment assessment of movement behaviors [25]. With the possibility of capturing the full continuum of movement behaviors using wearable accelerometry, there has been a conceptual shift and general consensus to use a more holistic analytical approach to evaluate associations involving all 24‐h movement behaviors in epidemiological studies [18, 26]. This paradigm shift moves away from examining each component part of 24‐h movement behaviors as independent exposures, so that the combined and joint associations of these behaviors on health markers could be assessed [18, 26]. This is because movement behaviors are mutually exclusive components of a finite 24‐h day and are inherently compositional, whereby time use is constrained to 24 h [19, 21, 27]. Proper examination of the associations between 24‐h movement behaviors and health outcomes, thus, requires analytical approaches that account for these compositional properties and the interdependent nature of time‐use data [18, 19, 26].

One suitable analytical technique for analyzing the 24‐h movement behavior data is compositional data analysis (CoDA), which has been increasingly used in 24‐h movement behavior research [26–28]. CoDA respects the characteristics of time‐use data, accounting for the fact that a change of time spent on one component of the 24‐h movement behavior would lead to an equal amount of time reallocated to one or a combination of other component parts [26–28]. Importantly, CoDA also enables the modeling of theoretical time reallocations, allowing researchers to examine how redistributing time among components of the 24‐h day may influence health outcomes [26–30].

Several prior studies have used CoDA to examine the associations between accelerometer‐determined 24‐h movement behaviors and mental health risks in adults, mainly assessing the symptoms of depression [14, 31–33]. Evidence from earlier research involving middle‐aged adults (46‐years‐old) and accelerometer‐measured movement behaviors indicated that spending more time in MVPA, relative to other behaviors, was associated with lower depressive symptoms [31]. In another CoDA study consisting of adults and older adults (mean age 47 years), SB was shown to be detrimentally associated with depressive symptoms [32]. Similarly, SB is also considered a risk factor for increased depression and anxiety symptoms in adults (mean age 56 years) [14]. Moreover, a recent systematic and meta‐analysis has suggested that reallocating time from SB to physical activity was significantly associated with reductions in depression risk for the adult subgroup [33]. These suggest that replacing SB by engaging in more MVPA may be the most effective time reallocation for improving mental health outcomes [14, 31–33]. However, less is known about whether and how other components of movement behaviors are linked to depressive symptoms and, in particular, to anxiety symptoms. Therefore, more research is needed to better understand how the components of 24‐h movement behaviors relate to depressive and anxiety symptoms when accounting for the time spent in other behaviors.

This cross‐sectional study used a CoDA approach (1) to examine the associations of 24‐h movement behaviors (sleep, SB, LPA, and MVPA) with depressive and anxiety symptoms and (2) to investigate time reallocations among 24‐h movement behaviors on depressive and anxiety symptoms.

## 2. Material and Methods

### 2.1. Study Material and Data Collection

All the study material in this study were derived from the Northern Finland Birth Cohort 1966 (NFBC1966) [34]. The NFBC1966 birth cohort study originally comprised of 12,058 live‐born children (over the entire year in 1966) across the northernmost provinces of Finland (Oulu and Lapland). Since birth, participants have been followed up on a regular basis at the ages of 1, 14, 31, and 46 years.

The present study involved data collected in the latest follow‐up performed when the participants were 46‐years‐old. In 2012, all cohort members who were alive and residing in Finland with a known address (*N* = 10,331) were invited to participate in the follow‐up study that consisted of both self‐reported questionnaires and clinical examination. The questionnaires cover information on personal background, lifestyle factors, socioeconomic status, employment, household incomes, and various health indicators, including the assessment of depressive and anxiety symptoms using three different instruments. Detailed information on the NFBC1966 study, including the 46‐year follow‐up, can be found elsewhere [35].

Written informed consent was obtained from all NFBC1966 participants, and the study was conducted in accordance with the Declaration of Helsinki. The NFBC1966 46‐year follow‐up study was approved by the Ethical Committee of the Northern Ostrobothnia Hospital District in Oulu, Finland (94/2011). Data was processed following the European Union general data protection regulation (679/2016) and the Finnish Data Protection Act.

#### 2.1.1. Assessing Depressive and Anxiety Symptoms

##### 2.1.1.1. Beck Depression Inventory‐II (BDI‐II)

The BDI‐II questionnaire is the revised version of the original BDI introduced in 1961 [36]. The BDI‐II is a 21‐item self‐reported questionnaire on the key symptoms of depression used for the assessment of depression intensity, with a clinical sensitivity of 0.92 [37]. The BDI‐II shows high internal consistency (*α* = 0.9), with a moderate to high retest reliability, which ranged between 0.76 and 0.93 [38]. For each item, four statements about a particular depressive symptom are presented in increasing severity, whereby the responder needs to select one statement for each item. The final scoring for the severity of depressive symptoms was calculated by summing up the number of points allocated to each statement, totaling to a possible maximum score of 63. A total score of 13 and less is classified as minimal depressive symptoms, 14–19 as mild, 20–28 as moderate, and 29–63 as severe [39].

##### 2.1.1.2. Generalized Anxiety Disorder (GAD‐7) Assessment

The GAD‐7 assessment is one of the screening tools available to assess the severity of anxiety disorders [40]. The GAD‐7 tool is a rapid and efficient initial screening for individuals to determine their anxiety symptoms, with high sensitivity (89%) and specificity (82%) [40]. The GAD‐7 tool has a high internal consistency (*α* = 0.89) and demonstrated good reliability [41]. The self‐reported GAD‐7 questionnaire comprises of seven items, covering the main symptoms of anxiety disorders. Responders were required to answer the questionnaire depending on the frequency of each symptom occurrence over the past 2 weeks, which ranged from “Not at all” (0 point) to “Nearly every day” (3 points).

The responses from each question were rescaled to ensure consistency with the original GAD‐7 questionnaire. The final GAD‐7 score was derived by summing up the responses for each question, with a possible maximum score of 21. A total score of 0–4 is classified as minimal anxiety symptoms, 5–9 as mild, 10–14 as moderate, and 15–21 as severe [40].

##### 2.1.1.3. Hopkins Symptom Checklist‐25 (HSCL‐25)

The HSCL‐25 questionnaire consisted of 25 items—10 items related to anxiety symptoms and 15 items related to depressive symptoms, providing the possibility to use the depressive and anxiety symptoms subscales separately [42]. As a reliable tool with good validity [43, 44], the self‐reported HSCL‐25 questionnaire has a sensitivity of 76% and a specificity of 73% [45]. Responders were required to answer the questionnaire depending on the frequency of each symptom/feeling occurrence within the past week, which ranged from “Not at all” (0 point) to “Very much” (4 points). The mean HSCL‐25 scores were obtained for all participants by averaging the responses for each question, resulting in a value between the range of 0 to a possible maximum score of 4. A recommended cut‐off score of 1.75 is commonly used to determine the presence of depressive and anxiety symptoms [45].

#### 2.1.2. Accelerometer‐Measured Waking Activities and Self‐Reported Sleep Duration

After the completion of health questionnaires, participants that agreed to participate in the study were instructed to wear the triaxial accelerometer (Hookie AM20; Traxmeet Ltd.) on the right‐side of their hip during all of their waking hours for 14 consecutive days, except when taking a bath or participating in water‐based activities. The accelerometer did not provide any feedback to the user. The raw acceleration signals from the accelerometer were collected and stored at 100 Hz.

Raw accelerometer signals were segmented into 6‐second non‐overlapping windows, and mean amplitude deviation (MAD) was computed for each window [46–48]. From the 6‐s time windows, non‐wear time was identified and excluded from analysis through a previously validated approach with a window size of 30 s to better handle artifactual acceleration [49]. The detected wear‐time intervals were then cross‐referenced with self‐reported time in bed—captured with two questions: “At what time do you normally go to bed?” and “At what time do you normally get out of bed?” All wear‐time periods that overlapped with a time in bed intervals were discarded.

The MAD values of the resultant acceleration, obtained from the Hookie AM20 accelerometer device, have been found to be a valid indicator of energy consumption during locomotion [46]. For those windows that were marked as wear time, the MAD values computed over 6‐s windows were converted to estimate energy expenditure expressed in metabolic equivalent of tasks (METs), defined as 3.5 mL/kg/min of oxygen consumption, using previously developed and validated prediction models [46]. The 6‐s windows were then classified into SB (METs <1.5), LPA (1.5–3.0 METs), and MVPA (METs >3.0).

A previously validated method for posture detection [50] was used to further differentiate between standing still and sitting or lying, as standing still should not be considered SB according to consensus definitions [51]. This method enables posture estimation from hip‐based raw acceleration data by leveraging the constant earth’s gravity vector and upright walking posture. It has demonstrated good to excellent accuracy compared with thigh‐worn posture classification as the ground truth under free‐living conditions [50].

Participants were required to provide four or more valid days of accelerometry, with each valid day defined as ≥10 h of monitor wear time. The criterion of at least 4 days with a minimum of 10 h of wear time has been commonly used in the literature, which provides an overall acceptable estimate of physical activity behaviors [25]. The average daily duration (min/day) spent in each movement intensity (SB, LPA, and MVPA) was calculated by summing the total time spent in each intensity across all valid days and dividing by the number of valid days. For the purposes of this study, LPA constituted the sum of all minutes per day spent standing still and in LPA.

**Figure 1:**
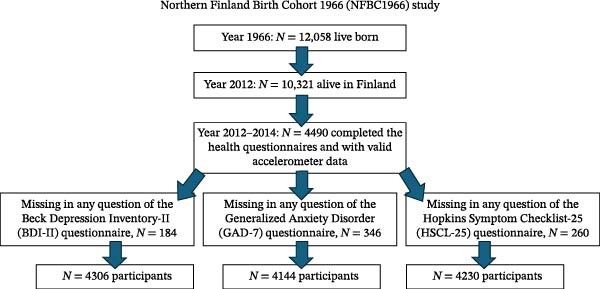
Flowchart on the selection of study participants from the NFBC1966 dataset in the analysis.

Information on sleeping duration was obtained from the NFBC1966 questionnaires completed by the participants, specifically “How many hours do you sleep on average at night?,” which was converted into minutes per day. A schematic representation of the eligible NFBC1966 participants included in this study is shown in Figure 1.

#### 2.1.3. Covariates

Potential confounders were selected a priori according to previous research [52, 53]. They include gender, employment status, smoking, education level, alcohol consumption, household income, and marital status. Participants who are entrepreneurs or self‐employed were considered to be employed. The alcohol intake of participants (g/day) was calculated from several items evaluating drinking habits [54]. Participants who smoke at least once a week were classified as smokers.

Unmarried, divorced, and widowed participants were considered single for their marital status. Self‐reported annual household income and educational attainment was used as a proxy for socioeconomic status in this study. The educational attainment of participants was grouped according to the Finnish educational system—no vocational training, vocational training (including college‐level training), and university degree. Only the highest educational attainment that has been completed at the point when filling in the questionnaire was taken into consideration for this study.

### 2.2. Statistical Analysis

Descriptive statistical analysis was performed on the outcome variables and covariates, specifically unpaired *t*‐tests (for means) and chi‐square tests (for categories). Graphical visualization (histograms or bar plots) of each outcome variable showed a highly right‐skewed distribution, with a large number of zeros for outcomes measured by BDI‐II and GAD‐7. In order to satisfy the assumption of normality, the Box–Cox transformation was applied to transform all the outcome variables. The Box–Cox transformation is a method to transform skewed distributed outcome variables into normal distribution [55]. Based on the optimal lambda values generated, BDI‐II and GAD‐7 scores were log‐transformed, while the HSCL‐25 mean scores were transformed using the power transformation. Statistical significance was determined by a *p*‐value of less than 0.05.

CoDA was used to examine the associations between 24‐h movement behaviors with depressive and anxiety symptoms, assessed by BDI‐II, GAD‐7, and HSCL‐25. Compositional data is a type of constrained data (in proportions or percentages) with a fixed constant sum constraint [21, 27, 28]. Time spent in sleep, SB, and physical activities are compositional data in nature because all component parts of the 24‐h movement behaviors sum up to 1440 min in a day. Due to this constraint, compositional explanatory variables cannot be directly included in linear regression models [56]. Following the guidelines for CoDA with movement behavior data [27, 28], the movement behavior composition for each participant was expressed as ratios of its parts using isometric log‐ratio (ilr) transformations before the regression analyses.

Isometric log‐ratio where the relative information of time spent between different movement behavior components could be represented by the following triplet formula [21, 27, 57]:

z1=34lnMVPALPA×SB×Sleep3;  z2=23lnLPASB×Sleep;  z3=12lnSBSleep.

The average duration per day spent in MVPA, LPA and SB was obtained (in minutes) after the classification of accelerometer data, whereas the average sleep duration in minutes per day was directly retrieved from the questionnaire. Thereafter, the geometric mean of each movement behavior was rescaled so that the total summed up to 1440 min (or 24 h) for each participant [27, 47]. The sleep duration, together with time spent in SB, LPA, and MVPA, were then transformed into ilr‐coordinates for each participant.

Complete case analysis was used in the compositional regression models, and all three ilr‐coordinates were entered as independent variables together with the chosen covariates. Three ilr‐coordinates (*Z*_1_, *Z*_2_, and *Z*_3_) were used to represent each behavior from the four‐part composition. For example, the relative importance of MVPA relative to the geometric mean of the other component parts of movement behaviors, such as LPA, SB, and sleep, was represented by ilr‐coordinate *Z*_1_. Therefore, four different triplets, including the triplet formula shown above, were obtained from different combinations of 24‐h movement behaviors.

The ilr values indicate the log‐ratio information of one component part relative to other component parts of 24‐h movement behaviors. As the ilr values contain only relative information, the effect sizes of the associations cannot be intuitively interpreted [21, 27, 28]. In accordance with the published CoDA guidelines [27, 28], time reallocations between movement behaviors were estimated to facilitate meaningful interpretation of their associations with the health outcomes. In the time reallocation analysis, changes of up to 30 min in movement behavior composition were examined, as this duration represents a realistic and achievable modification of movement behavior within our study population. The average movement behavior composition was used as the reference. Percentage differences in outcomes relative to the population mean of the respective outcome variables were then calculated.

The dataset was processed using the Statistical Package for the Social Sciences (SPSS, version 29) by IBM. Compositional regression analysis on all the different models was performed using the R software (version 4.3.2) and RStudio (version 2023.09.1+494) by Posit. The “compositions” R package was used for transforming the Aitchison compositional data into ilr‐coordinates.

## 3. Results

### 3.1. Descriptive Statistics

In this study, 4490 participants had valid accelerometry data, together with all questionnaires and measurement data required for the analysis. Participants with valid accelerometry data wore the accelerometer for 12.8 days on average, spent on average 450 min/day in sleep, 435 min/day in SB, 342 min/day in LPA, and 45 min/day in MVPA. Among participants with valid accelerometry data, 57.2% were female, 87.4% were employed, 16.1% were smokers, and the average alcohol consumption was 10.43 g per day. In addition, 10.6% had a high annual household income, and 28.6% completed higher education. Full descriptive statistics of the variables between the participants with valid accelerometry data and participants without valid accelerometry data are tabulated in Table 1.

**Table 1:** Characteristics of the participants with and without valid accelerometry data.

Variable	With valid accelerometry data (*N* = 4490)	Without valid accelerometry data (*N* = 2657)
**Gender** ^∗^		
Male	1921 (42.8%)	1378 (51.9%)
Female	2569 (57.2%)	1279 (48.1%)

**Education** ^∗^		
No vocational training	127 (2.8%)	129 (4.9%)
Vocational training	2941 (65.5%)	1581 (59.5%)
Higher education	1285 (28.6%)	541 (20.4%)
Missing	137 (3.1%)	406 (15.3%)

**Employment** ^∗^		
Employed	3924 (87.4%)	1897 (71.4%)
Unemployed	487 (10.8%)	387 (14.6%)
Missing	79 (1.8%)	373 (14.0%)

**Marital status** ^∗^		
Married/cohabiting/registered partnership	3564 (79.4%)	1690 (63.6%)
Single	911 (20.3%)	631 (23.7%)
Missing	15 (0.3%)	336 (12.6%)

**Household income** ^∗^		
Low (≤50,000 €/year)	1674 (37.3%)	1009 (38.0%)
Medium (50,001–100,000 €/year)	1892 (42.1%)	812 (30.6%)
High (>100,000 €/year)	476 (10.6%)	207 (7.8%)
Missing	448 (10.0%)	629 (23.7%)

**Smoking status** ^∗^		
Non‐smoker	3480 (77.5%)	1571 (59.1%)
Smoker	721 (16.1%)	626 (23.6%)
Missing	289 (6.4%)	460 (17.3%)

**Alcohol intake** ^∗^ (g/day)	10.43 (16.88)	13.44 (24.35)
Missing	3 (0.1%)	338 (12.7%)

**24-h movement behavior**		
Sleep (min/day)	450.14 (55.05)	447.32 (65.56)
Missing	—	364 (13.7%)
SB (min/day)	435.36 (89.05)	—
LPA (min/day)	341.60 (81.67)	—
MVPA (min/day)	45.37 (24.63)	—

*Note*: Mean and standard deviation (in brackets) are displayed for continuous variables, while counts and percentages (in brackets) are shown for categorical variables. Variables with significant differences between participants with and without valid accelerometry data were denoted by  ^∗^ for *p*  < 0.05.

Sex‐specific compositional means of different component parts within the 24‐h movement behaviors in comparison to the overall population mean are presented in Figure S1. Male participants engaged in more SB and MVPA than female participants, whereas female participants spent more time in LPA and had a longer self‐reported sleep duration than male participants. Amongst the different 24‐h movement behaviors, MVPA had the largest relative log‐ratio difference between male and female participants.

Descriptive statistics for the depressive and anxiety symptoms are compiled in Table 2. Among the NFBC1966 participants who completed the respective self‐reported questionnaires, significant differences in all the outcomes were observed between participants with valid accelerometry data and those without.

**Table 2:** Descriptive statistics of depressive and anxiety symptoms for NFBC1966 participants.

Symptom severity groupings	All participants	Participants with valid accelerometry data	Participants without valid accelerometry data
**Depressive symptoms by B** **DI-II** ^∗^ **(** ***N*** **)**	5860	4306	1554
Total BDI‐II score	5.55 (6.35)	5.31 (6.07)	6.21 (7.04)
Minimal symptoms (0–13)	5266 (89.9%)	3907 (90.7%)	1359 (87.5%)
Mild symptoms (14–19)	353 (6.0%)	247 (5.7%)	106 (6.8%)
Moderate symptoms (20–28)	170 (2.9%)	107 (2.5%)	63 (4.1%)
Severe symptoms (29–63)	71 (1.2%)	45 (1.0%)	26 (1.7%)
**Anxiety symptoms by GAD-7** ^∗^ **(** ***N*** **)**	5539	4144	1395
Total GAD‐7 score	2.49 (3.16)	2.40 (3.01)	2.76 (3.54)
Minimal symptoms (0–4)	4367 (78.8%)	3285 (79.3%)	1082 (77.6%)
Mild symptoms (5–9)	970 (17.5%)	734 (17.7%)	236 (16.9%)
Moderate symptoms (10–14)	146 (2.6%)	95 (2.3%)	51 (3.7%)
Severe symptoms (15–21)	56 (1.0%)	30 (0.7%)	26 (1.9%)
**Depressive and anxiety symptoms by HSCL-25** ^∗^ **(** ***N*** **)**	6414	4230	2184
Mean HSCL‐25 score	1.34 (0.34)	1.32 (0.32)	1.36 (0.38)
No symptoms (1–<1.75)	5701 (88.9%)	3803 (89.9%)	1898 (86.9%)
With symptoms (≥1.75–4)	713 (11.1%)	427 (10.1%)	286 (13.1%)

*Note*: Only participants who answered the entire depressive and anxiety symptoms questionnaire are included. Scores for depressive and anxiety symptoms were presented as mean with standard deviations in brackets. The categorical groupings of depressive and anxiety symptoms were presented as counts with percentages in brackets. Outcome variables with significant differences between participants with and without valid accelerometry data were denoted by  ^∗^ for *p*  < 0.05.

From the BDI‐II and GAD‐7 questionnaires, 90.7% and 79.3% of participants with valid accelerometry data had minimal depressive and anxiety symptoms, respectively. In comparison, only 1.0% of the participants had severe depressive symptoms, and only 0.7% had severe anxiety symptoms. Using a recommended cut‐off score of 1.75 in the HSCL‐25 questionnaire, 10.1% of the participants had depressive and anxiety symptoms, while 89.9% of the participants were considered to not have depressive and anxiety symptoms.

### 3.2. Compositional Regression Analysis

The results of the compositional regression analysis of the association between 24‐h movement behaviors with depressive and anxiety symptoms are displayed in Table 3. More time in MVPA and sleep was beneficially associated with depressive and anxiety symptoms, whereas more time in LPA and SB was negatively associated with depressive and anxiety symptoms. The same pattern of associations was observed for all three types of models in the three different tools used. All the 24‐h movement behaviors were significantly associated with the respective outcome variables in the fully adjusted model, except for the association between LPA and depressive symptoms measured by the BDI‐II tool.

**Table 3:** Compositional regression analysis of the association (estimates provided with 95% confidence intervals) between 24‐h movement behaviors with depressive and anxiety symptoms.

Model	24‐h movement behavior							
	Sleep		Sedentary		LPA		MVPA	
	Estimate (95% CI)	*p*‐Value	Estimate (95% CI)	*p*‐Value	Estimate (95% CI)	*p*‐Value	Estimate (95% CI)	*p*‐Value
**Depressive symptoms by BDI-II**								
Unadjusted (*N* = 4306)	−0.08 (−0.27 to 0.11)	0.41	**0.22** **(0.09 to 0.35)**	**<0.01**	0.11 (−0.01 to 0.22)	0.07	**−0.24** **(−0.30 to −0.19)**	**<0.01**
Adjusted for gender (*N* = 4306)	−0.17 (−0.37 to 0.02)	0.08	**0.30** **(0.17 to 0.43)**	**<0.01**	0.08 (−0.04 to 0.20)	0.18	**−0.21** **(−0.26 to −0.15)**	**<0.01**
Fully adjusted (*N* = 3500)	**−0.32** **(−0.54 to −0.11)**	**<0.01**	**0.40** **(0.25 to 0.54)**	**<0.01**	0.08 (−0.05 to 0.22)	0.23	**−0.16** **(−0.22 to −0.09)**	**<0.01**
**Anxiety symptoms by GAD-7**								
Unadjusted (*N* = 4144)	−0.17 (−0.35 to 0.00)	0.05	**0.15** **(0.03 to 0.26)**	**0.01**	**0.14** **(0.04 to 0.25)**	**<0.01**	**−0.11** **(−0.16 to −0.07)**	**<0.01**
Adjusted for gender (*N* = 4144)	**−0.27** **(−0.45 to −0.10)**	**<0.01**	**0.24** **(0.12 to 0.35)**	**<0.01**	**0.11** **(0.01 to 0.22)**	**0.03**	**−0.08** **(−0.12 to −0.03)**	**<0.01**
Fully adjusted (*N* = 3389)	**−0.33** **(−0.53 to −0.13)**	**<0.01**	**0.27** **(0.13 to 0.40)**	**<0.01**	**0.13** **(0.01 to 0.26)**	**0.03**	**−0.07** **(−0.13 to −0.02)**	**0.01**
**Depressive and Anxiety symptoms by HSCL-25**								
Unadjusted (*N* = 4230)	**−0.03** **(−0.05 to −0.01)**	**0.02**	**0.03** **(0.01 to 0.05)**	**<0.01**	**0.02** **(0.01 to 0.04)**	**<0.01**	**−0.02** **(−0.03 to −0.02)**	**<0.01**
Adjusted for gender (*N* = 4230)	**−0.04** **(−0.06 to −0.02)**	**<0.01**	**0.04** **(0.02 to 0.06)**	**<0.01**	**0.02** **(0.00 to 0.03)**	**0.01**	**−0.02** **(−0.03 to −0.01)**	**<0.01**
Fully adjusted (*N* = 3437)	**−0.05** **(−0.08 to −0.03)**	**<0.01**	**0.05** **(0.03 to 0.07)**	**<0.01**	**0.02** **(0.00 to 0.04)**	**0.02**	**−0.01** **(−0.02 to −0.01)**	**<0.01**

*Note:* Box–Cox transformation has been utilized for the outcome variables with *λ* = 0, 0, and −2 for BDI‐II, GAD‐7, and HSCL‐25, respectively. The fully adjusted model includes gender, employment status, smoking, education level, alcohol consumption, household income, and marital status. Estimates, confidence intervals and *p*‐values are rounded off to 2 decimal places. Significant estimate values are in bold for *p*  < 0.05.

### 3.3. Time Reallocation Analysis

**Figure 2:**
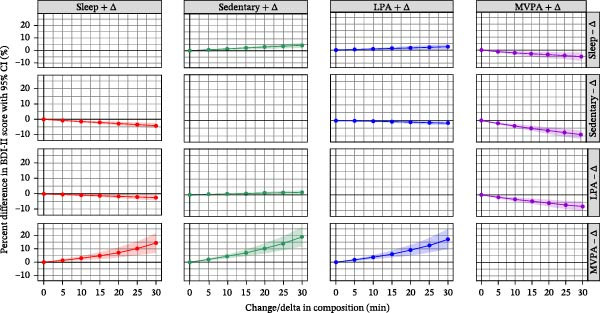
Predicted %difference in BDI‐II score for depressive symptoms (with 95% confidence intervals) based on the change in composition on the 24‐h movement behaviors (up to ± 30 min) in the fully adjusted model with BDI‐II score, using the population mean composition as reference. The fully adjusted model includes gender, employment status, smoking, education level, alcohol consumption, household income, and marital status.

The estimated differences of 24‐h movement behaviors on depressive symptoms are shown in Figure 2. The largest percentage difference in BDI‐II score was observed for changes in MVPA duration. For instance, 30 min reallocation from SB to MVPA was associated with a 9.0% (95% confidence interval [CI]: 6.3%, 11.6%) lower BDI‐II score. Moreover, 30 min reallocation from both sleep and LPA individually to MVPA was associated with 5.0% (95% CI: 1.9%, 8.1%) and 7.4% (95% CI: 4.3%, 10.5%) lower BDI‐II scores, respectively. Conversely, higher BDI‐II scores (by ~15%–20%) were associated with 30 min time reallocation from MVPA to each of sleep, SB, and LPA separately (Figure 2). All the other time reallocations between the 24‐h movement behaviors were only associated with very small differences (less than 5%) in the BDI‐II scores.

**Figure 3:**
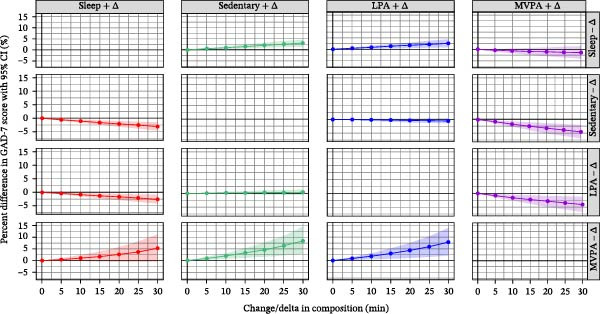
Predicted %difference in GAD‐7 score for anxiety symptoms (with 95% confidence intervals) based on the change in composition on the 24‐h movement behaviors (up to ± 30 min) in the fully adjusted model with GAD‐7 score, using the population mean composition as reference. The fully adjusted model includes gender, employment status, smoking, education level, alcohol consumption, household income, and marital status.

The predicted percentage difference in GAD‐7 score after time reallocation of the different 24‐h movement behaviors is illustrated in Figure 3. The largest percentage difference in GAD‐7 scores was observed for changes in the duration of MVPA. For example, the reallocation of 30 min from both SB and LPA individually to MVPA was associated with 4.5% (95% CI: 2.1%, 6.9%) and 4.0% (95% CI: 1.2%, 6.7%) lower GAD‐7 scores, respectively. In the other direction, when 30 min was reallocated from MVPA to SB and LPA separately, opposite patterns were observed, but with wider 95% CI ranges. The predicted percentage difference for GAD‐7 scores from the time reallocation in both directions between sleep and MVPA as well as between SB and LPA were not significant. Only very small differences (less than 5%) in the GAD‐7 scores were associated with all other time reallocations between the 24‐h movement behaviors.

**Figure 4:**
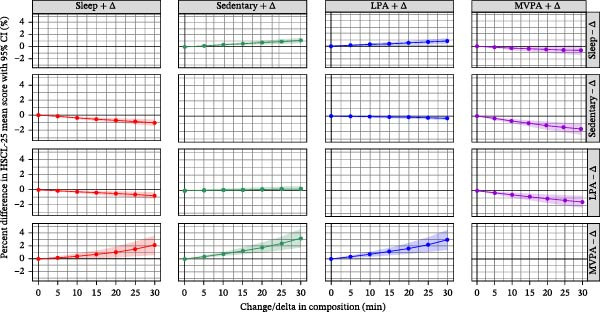
Predicted %difference in HSCL‐25 mean score for depressive and anxiety symptoms (with 95% confidence intervals) based on the change in composition on the 24‐h movement behaviors (up to ± 30 min) in the fully adjusted model with HSCL‐25 mean score, using the population mean composition as reference. The fully adjusted model includes gender, employment status, smoking, education level, alcohol consumption, household income, and marital status.

The predicted percentage difference from time reallocation analysis involving the HSCL‐25 mean score for depressive and anxiety symptoms is depicted in Figure 4. The reallocation of 30 min from SB and LPA individually to MVPA was associated with 1.7% (95% CI: 1.0%, 2.4%) and 1.5% (95% CI: 0.7%, 2.2%) lower HSCL‐25 mean scores, respectively. However, when 30 min was reallocated from MVPA to sleep, SB, and LPA separately, different trends were noticed, such as greater percentage difference in the HSCL‐25 mean scores, together with wider 95% CI ranges. In addition, for all other time reallocations not involving MVPA, only very small differences (less than 1%) in the HSCL‐25 mean scores were associated.

## 4. Discussion

The aim of this study is to investigate the associations of 24‐h movement behaviors with depressive and anxiety symptoms among middle‐aged adults using a CoDA approach. Depressive and anxiety symptoms were assessed with three different self‐reported questionnaires, including BDI‐II, GAD‐7, and HSCL‐25. The main finding of this study is that the composition of 24‐h time use is associated with depressive and anxiety symptoms. While more time spent in MVPA and sleep was consistently found to be associated with lower levels of depressive and anxiety symptoms, more time spent in SB and LPA was found to be associated with higher levels of depressive and anxiety symptoms. After adjustment for potential confounders, most favorable differences in depressive and anxiety symptoms were observed when time from sleep, SB, or LPA was reallocated to MVPA.

More time in MVPA, relative to all other behaviors, was favorably associated with depressive and anxiety symptoms. Reallocating time to MVPA from any other movement behaviors was associated with most favorable differences in depressive and anxiety symptoms. Previously, both experimental and observational studies have consistently demonstrated that MVPA is associated with lower risk of depression and anxiety [14, 31, 58, 59]. Similar findings have also been reported in studies examining the associations of 24‐h movement behaviors with symptoms of depression and anxiety using CoDA [14, 31, 32]. The underlying mechanisms by which MVPA may lower the symptoms of depression and anxiety are not fully understood. Several hypotheses have been proposed including physiological, psychological, and cognitive‐behavioral aspects, indicating a complex causal pathway [60]. Our study provides further support for the existing literature suggesting that MVPA is the most beneficial intensity of movement for depressive and anxiety symptoms, even after properly accounting for time spent in other 24‐h movement behavior components. Collectively, the findings of this study underscore the importance of engaging in more physical activity, especially MVPA, even at small doses, to lower depressive and anxiety symptoms.

The compositional regression analysis showed that LPA is unfavorably associated with depressive and anxiety symptoms as assessed by GAD‐7 and HSCL‐25. The association of LPA with depressive symptoms was not significant when assessed with BDI‐II. Modeling of time reallocation revealed that replacing SB with LPA could provide some benefits for depressive and anxiety symptoms, although the differences were marginal. The associations of LPA with anxiety symptoms have been less studied, in comparison to depressive symptoms. One previous study showed that reallocating time from SB to LPA is associated with higher anxiety symptoms instead [14]. Compared to MVPA, LPA is an accessible movement intensity for people who cannot engage in movement of higher intensities for different reasons, such as time constraints or physical limitations. LPA has been shown to provide several potential health benefits beyond mental health [47, 61, 62]. However, its associations with health outcomes appear to be outcome‐specific, and its potential benefits have consistently been reported to be less pronounced than those of MVPA.

Depressive symptoms were found to be associated with lower levels of physical activity and longer time in bed [63]. In contrast, increasing SB or LPA at the expense of sleep showed detrimental associations, and reallocating time from SB or LPA to sleep was still significantly associated with lower depressive and anxiety symptoms. These results highlight the importance of sleep on overall health and mental health, suggesting that doing more physical activity, despite being beneficial, should not be done at the expense of sleeping. The protective effect of sleeping on mental health is well‐documented, as research has mentioned that sleep duration is associated with mental health, including depression and anxiety [15, 64]. The beneficial association of sleep with depressive and anxiety symptoms is also consistent with earlier published studies [14, 32]. Nevertheless, it is important to consider the bidirectional relationship between sleep and mental health, in which depressive and anxiety symptoms can negatively affect the duration and quality of sleep [65, 66].

Overall, similar patterns were observed in the time reallocation analysis for depressive and anxiety symptoms across all screening tools. Our findings were mostly comparable to other similar studies involving 24‐h movement behaviors [67]. Even when studied in isolation, depressive symptoms were found to be associated with lower levels of physical activity [63]. In the same context, participants with depressive symptoms had spent significantly more time in SB, as well as less time in LPA and MVPA, than participants without depressive symptoms [68]. The difference in findings regarding the association of 24‐h movement behaviors with depressive and anxiety symptoms could potentially be due to the different age group or different screening tools used in comparison to previous studies [63, 67, 69]. Different approaches in accelerometry data collection, processing, or analysis might have also contributed to these contradictory findings.

Strengths of this research include the use of high‐quality data from a large‐scale population‐based cohort study covering different aspects of lifestyle factors. The severity of both depressive and anxiety symptoms was evaluated by multiple screening tools. Depressive symptoms were assessed by the BDI‐II tool and anxiety symptoms were measured by the GAD‐7 tool, whereas the HSCL‐25 tool was utilized to determine both depressive and anxiety symptoms. To our best knowledge, our study design is the first of its kind to incorporate different screening tools together with CoDA to determine how accelerometer‐measured SB, LPA, and MVPA and self‐reported sleep duration are associated with depressive and anxiety symptoms in middle‐aged adults. In our study, the compositional approach leveraged relative information amongst 24‐h movement behaviors in the analysis, allowing for better scrutiny of the inter‐relationships between each component. The choice of covariates was also carefully selected for analysis to avoid the issue of confounding. Another advantage of our study was that component parts of movement behaviors during waking hours were monitored with raw accelerometry, which has possibly provided a more precise estimate of physical activity intensities and SB [70]. Additionally, study participants wore the accelerometer for 14 consecutive days rather than the usual 7 consecutive days by other studies [71, 72]. This is a strength because it could provide a better representation of movement behaviors in the analysis.

Our study also has several drawbacks. The 24‐h movement behavior data from a population cohort was analyzed only in a single time period. The cross‐sectional design of the study inferred that the cause‐and‐effect relationship or reverse causality could not be determined. As data for this study was collected almost a decade ago, movement behaviors may be influenced by broader societal changes, including increased access to digital technologies and social media. Accelerometer‐measured waking activity behaviors were combined with self‐reported sleep duration to obtain the 24‐h time‐use. Given that sleep duration was self‐reported, the measurement accuracy for sleep is likely lower compared to the other movement behaviors. While self‐reported sleep durations are often higher than accelerometer‐measured sleep durations among middle‐aged adults, the differences between them are usually small [73]. Sleep duration did not include naps, which could be seen as a limitation. Participants meeting the inclusion criteria had lower depressive and anxiety symptoms and had some differences in other characteristics. Although the sample size with valid accelerometer data was relatively large (*N* = 4490), there could still be a risk for non‐response bias. Since the depressive and anxiety symptoms were assessed via questionnaires, there could also be a possibility of recall bias. Although different aspects of depressive and anxiety symptoms are covered in the screening tools, direct comparison between the tools should be made with caution due to the heterogeneity of mental health assessment tools [74]. Individuals, triggered by seasonal changes, may exhibit depressive and anxiety symptoms or the worsening of symptoms [75]. Although several studies had reported that people tend to feel more depressed during winter months [76], these seasonal effects were not incorporated into our analysis. In regions with uneven distribution of daylight hours due to seasonal variations, such as Finland, the amount of physical activity [77] and sleep [78, 79] in individuals may also vary across seasons. However, data collection of our study took place all‐year round between 2012 and 2014, thus, the effect of seasonality was not of great concern. The study sample consisting of NFBC1966 participants was homogenous in terms of age and ethnicity. Therefore, our findings may not be directly generalizable to individuals of other age groups or different ethnicities. The associations of 24‐h movement behaviors were examined without distinguishing between weekday and weekend day physical activity and SB. This is because health outcomes are primarily driven by the total volume of both physical activity and SB [11, 80]. Research indicates that individuals meeting recommended physical activity levels experience comparable health benefits regardless of whether the activity is distributed across the week or concentrated into fewer days (e.g., the “weekend warrior” pattern) [81]. Consequently, most national and international guidelines emphasize on the total duration and intensity of movement behaviors rather than specific timing or accumulation patterns. In contrast, variability in sleep duration across different days could be a factor related to depressive and anxiety symptoms [82]. However, as sleep duration was self‐reported in the present study, we were unable to examine weekday‐weekend differences in sleep patterns or assess how such variability may influence depressive and anxiety symptoms. The potential differences in how weekday versus weekend day movement behaviors relate to depressive and anxiety symptoms warrant further investigation.

## 5. Conclusions

Among the component parts of 24‐h movement behaviors, higher MVPA and sleep were found to be associated with lower depressive and anxiety symptoms. Time reallocation analysis revealed that substituting any component parts of 24‐h movement behaviors with MVPA had the most favorable association with depressive and anxiety symptoms. In contrast, reallocating time from SB to LPA was only marginally beneficial for depressive and anxiety symptoms. These findings emphasize the importance of MVPA, rather than LPA, for depressive and anxiety symptoms.

## Acknowledgments

The authors thank all NFBC1966 cohort participants and researchers who participated in the 46‐year follow‐up study. They acknowledge the NFBC project center for their contribution in managing the NFBC1966 cohort study.

## Funding

The present study has received funding from the Ministry of Education and Culture in Finland (Grants OKM/20/626/2022, OKM/76/626/2022, and OKM/68/626/2023) and the Research Council of Finland (DOACT project, Grant 361560; University of Oulu PROFI6 project, Grant 336449). The NFBC1966 received financial support from the University of Oulu (Grant 24000692), the Oulu University Hospital (Grant 24301140), and the European Regional Development Fund (Grant 539/2010 A31592). Open access publishing facilitated by Oulun yliopisto, as part of the Wiley ‐ FinELib agreement.

## Conflicts of Interest

The authors declare no conflicts of interest.

## Supporting Information

Additional supporting information can be found online in the Supporting Information section.

## Supporting information


**Supporting Information** Figure S1: Compositional means of different activities within the 24‐h movement behavior components in comparison to the overall population mean, separated by gender (*N* = 4490).

## Data Availability

The NFBC1966 datasets utilized in the current study are available from the University of Oulu, Infrastructure for Population Studies. Permission to use the data can be applied for research purposes via an electronic material request portal.
