# Supplementary material for: Compositional Associations of 24‐h Movement Behaviors With Depressive and Anxiety Symptoms in Middle‐Aged Adults
Source: Depress Anxiety. 2026 Apr 28;2026:6881070. doi: 10.1155/da/6881070 (PMC13123449; doi:10.1155/da/6881070)
Supplement: Supplementary file 1 — Supporting Information Figure S1: Compositional means of different activities within the 24‐h movement behavior components in comparison to the overall population mean, separated by gender (N = 4490). [file DA-2026-6881070-s001.docx]

Supplementary material


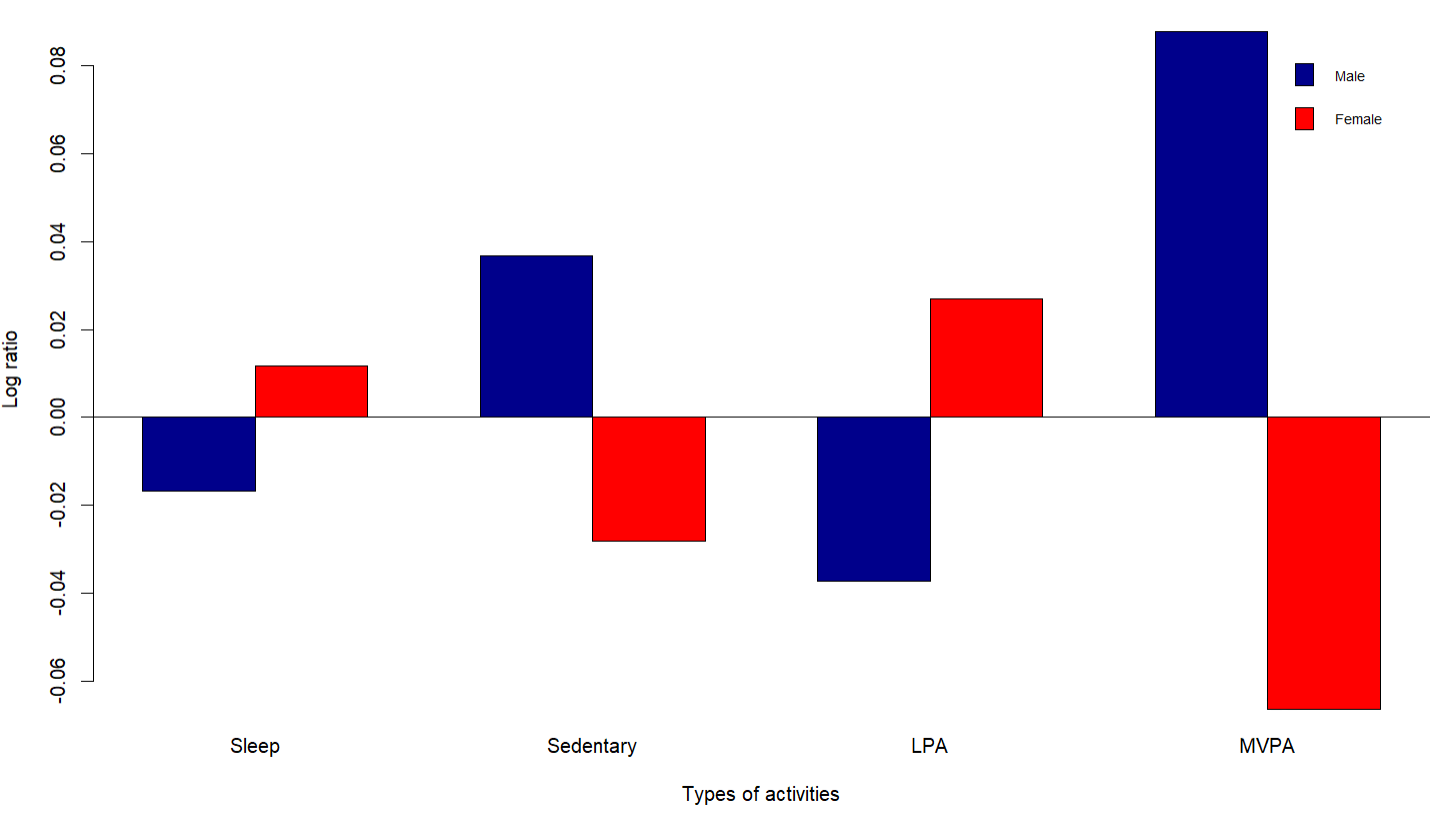


**Figure S1**: Compositional means of different activities within the 24-hour movement behavior components in comparison to the overall population mean, separated by gender (N=4,490).
